# Plasmidic *qnrA3* Enhances *Escherichia coli* Fitness in Absence of Antibiotic Exposure

**DOI:** 10.1371/journal.pone.0024552

**Published:** 2011-09-07

**Authors:** Adrien Michon, Nicolas Allou, Françoise Chau, Isabelle Podglajen, Bruno Fantin, Emmanuelle Cambau

**Affiliations:** 1 EA 3964, University Paris Diderot, Paris, France; 2 Microbiologie Hôpital Européen Georges Pompidou, APHP, Paris, France; 3 Medecine Interne Hôpital Beaujon, APHP, Clichy, France; 4 Bacteriologie, Groupe Hospitalier Saint Louis-Lariboisière-Fernand Widal, APHP, Paris, France; University of Birmingham, United Kingdom

## Abstract

The widespread presence of plasmid-mediated quinolone resistance determinants, particularly *qnr* genes, has become a current issue. By protecting DNA-gyrase from quinolones, Qnr proteins confer a low level quinolone resistance that is not sufficient to explain their emergence. Since Qnr proteins were hypothesized to act as DNA-binding protein regulators, *qnr* genes could have emerged by providing a selective advantage other than antibiotic resistance. We investigated host fitness of *Escherichia coli* isogenic strains after acquisition of the *qnrA3* gene, inserted either alone onto a small plasmid (pBR322), or harbored on a large conjugative native plasmid, pHe96(*qnrA3*) found in a clinical isolate. The isogenic strains were derived from the susceptible *E. coli* CFT073, a virulent B2 group strain known to infect bladder and kidneys in a mouse model of pyelonephritis. *In vitro* experiments included growth analysis by automatic spectrophotometry and flow cytometry, and competitions with CFU enumeration. *In vivo* experiments included infection with each strain and pairwise competitions in absence of antimicrobial exposure. As controls for our experiments we used mutations known to reduce fitness (*rpsL* K42N mutation) or to enhance fitness (*tetA* deletion in pBR322). *E. coli* CFT073 transformed with pBRAM(PBR322-*qnrA3*) had significantly higher maximal OD than *E. coli* CFT073 transformed with pBR322 or pBR322Δ*tetA*, and *in vivo* competitions were more often won by the *qnrA3* carrying strain (24 victories vs. 9 loss among 42 competitions, p = 0.001). In contrast, when pHe96(*qnrA3*) was introduced by conjugation in *E. coli* CFT073, it exerted a fitness cost shown by an impaired growth observed *in vitro* and *in vivo* and a majority of lost competitions (33/35, p<0.0001). In conclusion, *qnrA3* acquisition enhanced bacterial fitness, which may explain *qnr* emergence and suggests a regulation role of *qnr*. However, fitness was reduced when *qnrA3* was inserted onto multidrug-resistant plasmids and this can slow down its dissemination without antibiotic exposure.

## Introduction

Fluoroquinolones are antibacterial drugs that bind to type II topoisomerases (DNA gyrase and topoisomerase IV) and inhibit DNA re-ligation after enzyme cut [1], [2]. These drugs are very useful, especially for treatment of urinary tract infections due to *Enterobacteriaceae* [1]. Fluoroquinolone resistance rate has increased much for the last years and is mostly due to their large use [3], [4]. Classical mechanisms of resistance are chromosomal mutations in the genes encoding the quinolone targets or causing enhanced efflux [1], [5]. More recently, plasmid-mediated resistance determinants have been reported encoding for different proteins: the Qnr proteins which belong to the pentapeptide repeat family (PRP) [6], [7], the acetyltransferase AAC(6′)-Ib-cr [8] and the QepA active efflux pump [9]. *Enterobacteriaceae* with plasmid-mediated quinolone resistance (PMQR) due to *qnr* genes have been worldwide described with an increase in their prevalence [10]. This rapid widespread is surprising because the acquisition of a *qnr* gene only confers a low-level resistance to fluoroquinolones [6], [11], [12]. Although this low-level resistance can be clinically relevant and probably contributed to *qnr* dissemination [13], [14], this does not fully explain the emergence of the *qnr* genes.

In clinical strains, *qnr* genes were essentially found on multi-drug resistance (MDR) plasmids, but chromosomal *qnr* genes have been also described in environmental bacterial species that are the supposed reservoir of these genes [15]–[18]. The native function of Qnr proteins still remains unknown [10]. They bind to type II topoisomerases and thus protect them from quinolone binding and action [7], [19], [20]. Functional and crystallography analyses of PRPs closed to Qnr are in favor of a role of Qnr proteins in topoisomerase regulation [21]–[25].

Most antibiotic resistance mechanisms, particularly fluoroquinolone resistance mutations, are associated with a fitness cost [26]–[29]. However, fitness cost of horizontal transferable resistance genes is often compensated by the regulation of transcription factors encoded by other genes harbored onto the same plasmid [30]. Nonetheless, interplay between resistance and fitness are not always concordant, and bacteria can reverse the cost induced by resistance acquisition. Several mutations could provide both enhanced fitness and increased resistance [29], [31]. Emergence of resistance can be driven by Darwinian selection for improved fitness, and not only by the antibiotic use [26]. We hypothesized that Qnr proteins have an effect on bacterial growth and fitness, which may have contributed to the emergence of *qnr* genes in commensal bacteria.

The aim of our study was to evaluate the impact of the *qnr* gene acquisition on bacterial fitness. Therefore we compared the fitness of isogenic strains of *Escherichia coli* with and without the *qnrA3* gene, whether alone onto a small plasmid or carried onto a large conjugative multi-drug-resistant native plasmid. Growth and competitive performances were studied *in vitro* and *in vivo* using a mouse model of pyelonephritis.

## Results

### Description of the isogenic systems expressing *qnr* or not

Two systems of isogenic strains were derived from *E. coli* CFT073, a virulent strain belonging to the phylogenetic group B2 and whose genome has been sequenced [32]. This strain was originally used to set the murine model of pyelonephritis used in this study [33]. We selected a streptomycin resistant mutant (Sm^R^) of *E. coli* CFT073 in order to have a resistance marker for the recipient strain after acquisition of the plasmid pHe96, which is a multidrug resistant plasmid not mediating streptomycin resistance. This mutant was selected using 160 µg/ml streptomycin at a proportion of ca.10^−9^ and harbored a *rpsL* K42N mutation which is consistent with its high level of resistance (MIC>512 µg/ml) and the stability of this resistance. Although pHe96 contains an *ant3″-I* gene known to confer streptomycin resistance [34], this gene is truncated and we confirmed that pHe96 does not confer streptomycin resistance by transferring pHe96 into *E. coli* J53. The MIC of streptomycin was 4 mg/l for this transconjugant and was stable.

The first isogenic system included five strains: *E. coli* CFT073, *E. coli* CFT073 (pBR322) and *E. coli* CFT073 transformed with three other plasmids derived from pBR322 and described in Figure S1: pBRΔ*tetA* where the tetracycline resistance gene (*tetA*) was deleted, pBRAM1 where the *qnrA3* gene was cloned including the 24-bp DNA motif upstream from *qnrA3*, and pBRAM2 where *qnrA3* was cloned including the 233-bp DNA motif upstream. In both pBRAM1 and pBRAM2, *qnrA3* was inserted into pBR322 by inactivating the *tetA* gene. Minimal inhibitory concentrations (MIC) of quinolones performed on the five strains showed that *qnrA3* expressed quinolone resistance equally (Table 1) with an increase of 4-, 8-, 10- and 16-fold for nalidixic acid, ofloxacin, ciprofloxacin and norfloxacin, respectively.

**Table 1:** Minimal Inhibitory Concentrations (MIC) of quinolones against the strains of the two isogenic systems derived from *E. coli* CFT073.

*E. coli* strains	MIC^a^ (µg/ml)					
	NAL	NOR	OFX	CIP	AMK	TOB
*E. coli* CFT073	2	0.064	0.094	0.012	1.5	0.75
*E. coli* CFT073(pBR322)	2	0.064	0.094	0.012	1.5	0.5
*E. coli* CFT073(pBRΔ*tetA*)	2	0.064	0.094	0.012	1.5	0.5
*E. coli* CFT073(pBRAM1)	8	1	0.75	0.125	1	0.5
*E. coli* CFT073(pBRAM2)	8	1	0.75	0.125	1.5	0.5
*E. coli* CFT073-Sm^R^	2	0.064	0.094	0.012	1.5	0.75
*E. coli* CFT073-Sm^R^(pHe96)	6	3	0.75	0.75	48	32
*E. coli* CFT073-Sm^R^(pHe96) “R42”	6	3	0.75	0.75	48	32

a Minimal inhibitory concentrations measured by E-test for quinolones and aminoglycosides. NAL, nalidixic acid; NOR, norfloxacin; OFX, ofloxacin; CIP, ciprofloxacin; AMK, amikacin; TOB, tobramycin.

The second isogenic system included three strains: *E. coli* CFT073-Sm^R^, *E. coli* CFT073-Sm^R^(pHe96), and a variant of this transconjugant named *E. coli* CFT073-Sm^R^(pHe96) “R42”, obtained after one passage in the mouse and which showed improved growth *in vitro* and *in vivo* and higher plasmid stability (see below). The evolved variant “R42” had the same phenotype for antibiotic resistance than the original strain CFT073-Sm^R^(pHe96) including for streptomycin resistance. The acquisition of pHe96 conferred, as described previously [16], a 62- and 50-fold increase in the MIC of ciprofloxacin and norfloxacin (Table 1), respectively, because this plasmid harbored the *aac6′-Ib-cr* gene in addition to *qnrA3* [16]. In contrast, ofloxacin and nalidixic acid MICs were the same for the transconjugants CFT073-Sm^R^(pHe96) and for *E. coli* CFT073(pBRAM1) and *E. coli* CFT073(pBRAM2) confirming that *aac6′-Ib-cr* has no effect on these quinolones, and showing that the expression of *qnrA3* was similar whether it was harbored on the small plasmid derived from pBR322 or on the large clinical plasmid from which *qnrA3* originated [11], [16]. The “R42” variant showed similar sensitivity to quinolones as it parental strain.

Clinical *E. coli* isolates carrying a conjugative multidrug resistant plasmid harboring a *qnr* gene were also tested in the *in vitro* experiments along with the strain *E. coli* J53, a K-12 derivative used as a recipient strain for conjugation [35], [36]. Description of the strains, their *qnr* allele and the quinolone resistance conferred (increase in quinolone MICs observed for the transconjugants) was done previously [11], [13], [35], [37]. *E. coli* J53 transconjugants harboring *qnr*-positive plasmids were studied in similar *in vitro* experiments as were the two isogenic systems based on *E. coli* CFT073 (Table S1).

*E. coli* CFT073-Sm^R^ was used as a negative control for host fitness since it has been shown that streptomycin resistance, and especially the *rpsL* mutation K42N, had a fitness cost [26], [38], [39]. *E. coli* CFT073(pBRΔ*tetA*) was used as a positive control with regard to *E. coli* CFT073(pBR322) since the *tetA* gene has been shown to have a cost for growth when carried by pBR322 and constitutively expressed [40], [41].

### In vitro growth capacity of strains harboring *qnrA3*

In vitro growth capacity was measured by automated spectrophotometry for the calculation of the maximal growth rate, the doubling time and the maximal optical density, and by flow cytometry for the size of bacterial cells.

Growth parameters of *E. coli* CFT073 and its four isogenic strains harboring the plasmid pBR322 or one of its derivates (pBRΔ*tetA*, pBRAM1 and pBRAM2) are shown in Table 2. Maximal OD was significantly higher for all strains harboring a *qnrA3* carrying plasmid. This gain was not due to the inactivation of *tetA*, since the increase in maximal OD was significantly higher for *E. coli* CFT073 transconjugants harboring pBRAM1 or pBRAM2 (acquisition of *qnrA3* at the place of *tetA*) than for the transconjugant harboring pBRΔ*tetA*. No significant difference was seen in the doubling time between strains where *qnrA3* was present (*E. coli* CFT073 transformed with pBRAM1 or pBRAM2) and those where *qnrA3* was absent (*E. coli* CFT073 transformed with pBRΔ*tetA* or pBR322).

**Table 2:** *In vitro* growth parameters for the strains of the first isogenic system based on *E. coli* CFT073 carrying pBR322 derivatives harboring *qnrA3* or not.

*E. coli* strains	Maximal growth rate in log(OD)/h.		Doubling time in min		Maximal OD	
	mean	*p* a	mean	*p* a	mean	*p* a
CFT073(pBRΔ*tetA*)	0.96 (+/−0.02)	-	18.9 (+/−0.3)	-	1.07 (+/−0.02)	-
CFT073(pBR322)	0.94 (+/−0.02)	0.21	19.2(+/−0.3)	0.21	1.05 (+/−0.06)	0.6
CFT073(pBRAM1)	0.94 (+/−0.01)	0.17	19.2 (+/−0.3)	0.17	1.11 (+/−0.02)	0.005
CFT073(pBRAM2)	0.94 (+/−0.02)	0.13	19.2 (+/−0.5)	0.13	1.10 (+/−0.02)	0.01

Data obtained from automatic OD measurements (n = 15) in Trypticase Soy Broth and expressed as mean and confidence interval 95%.

a The comparison was performed with a Wilcoxon test between each strains and the corresponding control *qnr*-negative strain CFT073(pBRΔ*tetA*). *p*<0.05 was considered significant.

Acquisition of the plasmid pHe96 by *E. coli* CFT073-Sm^R^ was responsible for a significant lengthening of doubling time and decrease in maximal OD (Table 3), at the opposite of what was shown above for pBRAM plasmids. Similar results were obtained in brain heart infusion and minimal media (data not shown). The “R42” variant of *E. coli* CFT073-Sm^R^(pHe96) showed significantly better growth parameters than the original transconjugant strain but stayed below the control strain *E. coli* CFT073-Sm^R^. Comparing results between *E. coli* CFT073-Sm^R^ with its parental strain *E. coli* CFT073 confirmed that the streptomycin resistance due to the *rpsL* K42N mutation [38] was associated with a decrease in maximal growth rate (0.95 logOD/h+/−0.02 for *E. coli* CT073 vs. 0.7 logOD/h+/−0.01 for *E. coli* CFT073-Sm^R^) and increase in the doubling time (18.9 h+/−0.74 for *E. coli* CFT073 vs. 25.9 h+/−0.5 for *E. coli* CFT073-Sm^R^). Overall, the decrease in growth capacity conferred by the acquisition of pHe96 seemed to reduce fitness less than the *rpsL* mutation did.

**Table 3:** *In vitro* growth parameters for the strains of the second isogenic system based on acquisition of the multidrug resistant *qnrA3*-positive plasmid pHe96.

*E. coli* strains	Maximal growth rate in log(OD)/h.		Doubling time in min		Maximal OD	
	mean	*p* a	mean	mean	*p* a	mean
CFT073-Sm^R^	0.70 (+/−0.01)	-	25.9 (+/−0.5)	-	1.08 (+/−0.01)	-
CFT073-Sm^R^(pHe96)	0.67 (+/−0.02)	0.02	27.1 (+/−0.9)	0.02	0.94 (+/−0.03)	<0.0001
CFT073-Sm^R^(pHe96) R42	0.70 (+/−0.01)	0.8	26.1 (+/−0.8)	0.8^b^	0.97 (+/−0.02)	<0.0001

a The comparison was performed with a Wilcoxon test between each strains and the corresponding control *qnr*-negative strain CFT073-Sm^R^. *p*<0.05 was considered significant.

b The difference between the “R42” variant and *E. coli* CFT073-Sm^R^ (pHe96) was significant (p = 0.04).

**Figure 1:**
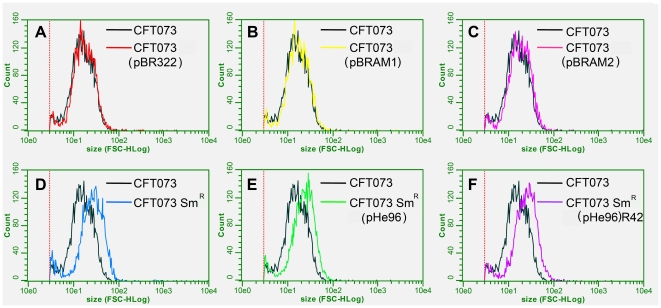
Cell size measured by flow cytometry for the strains of the two isogenic systems. Each graphic compares size measurement for bacterial cell populations consisting of the reference susceptible strain *E. coli* CFT073 (in black) and of the tested strain : *E. coli* CFT073(pBR322) (*qnr*-negative) in red (part A), *E. coli* CFT073(pBRAM1) (*qnrA3*-positive) in yellow (part B), *E. coli* CFT073(pBRAM2) (*qnrA3*-positive) in pink (part C), *E. coli* CFT073-Sm^R^ (*qnr*-negative) in blue (part D), *E. coli* CFT073-Sm^R^(pHe96) (*qnrA3*-positive) in green (part E) and its variant R42 in purple (part F). Measurements were made separately, not in a competitive assay.

Results (arbitrary unit with 95% CI) of flow cytometry are presented in the Figure 1. Sizes measured by flow cytometry were 20.6 [18.1–23.1], 19.1 [18–20.2] 19.9 [19–20.8], 20.6 [20–21.2] for the *E. coli* CFT073 wild type strain and its transformants with pBR322 (*tet*+, *qnr*−), with pBRAM1 (*tet*−, *qnr*+), and with pBRAM2 (*tet*−, *qnr*+), respectively. For *E. coli* CFT073-Sm^R^, its transconjugant with pHe96 and the variant isolate R42 of this transconjugant, sizes were 32.8 [30.8–34.8], 28.9 [26.8–31], and 30.1 [27.9–32.3], respectively, which were all 34.5% higher than those of *E. coli* CFT073. This showed that the acquisition of *qnrA3* onto pBRAM1 or pBRAM2 or onto pHe96 did not change significantly the size of bacteria, contrarily to the mutation of the recipient strain *E. coli* CFT073-Sm^R^, which was responsible for the increase in bacterial cell size.

### Enhanced fitness after acquisition of *qnrA3* onto pBR322 derived plasmids

*In vitro* competitive assays were run six times by cultivating *E. coli* CFT073(pBRAM1) (*qnrA3+*) or *E. coli* CFT073(pBRAM2) (*qnrA3+*) with *E. coli* CFT073(pBR322) (*tetA*+) or *E. coli* CFT073 in a 1∶1 ratio. Population increase was measured by CFU counting. Relative Fitness was calculated as the ratio of the increase of each population (see material and methods for details) [42]–[45]. Comparing with *E. coli* CFT073(pBR322), estimated Relative Fitness (RF) was close to 1 (1.03+/−0.19), meaning that none of the two competing strains had a selective advantage upon the other *in vitro*. Comparing with *E. coli* CFT073, RF was estimated at 1.12+/−0.2, showing that the entire plasmid had no significant biological cost. The mean plasmid loss was less than 3% after 30 generations for all the strains harboring pBR322-derivatives.

**Figure 2:**
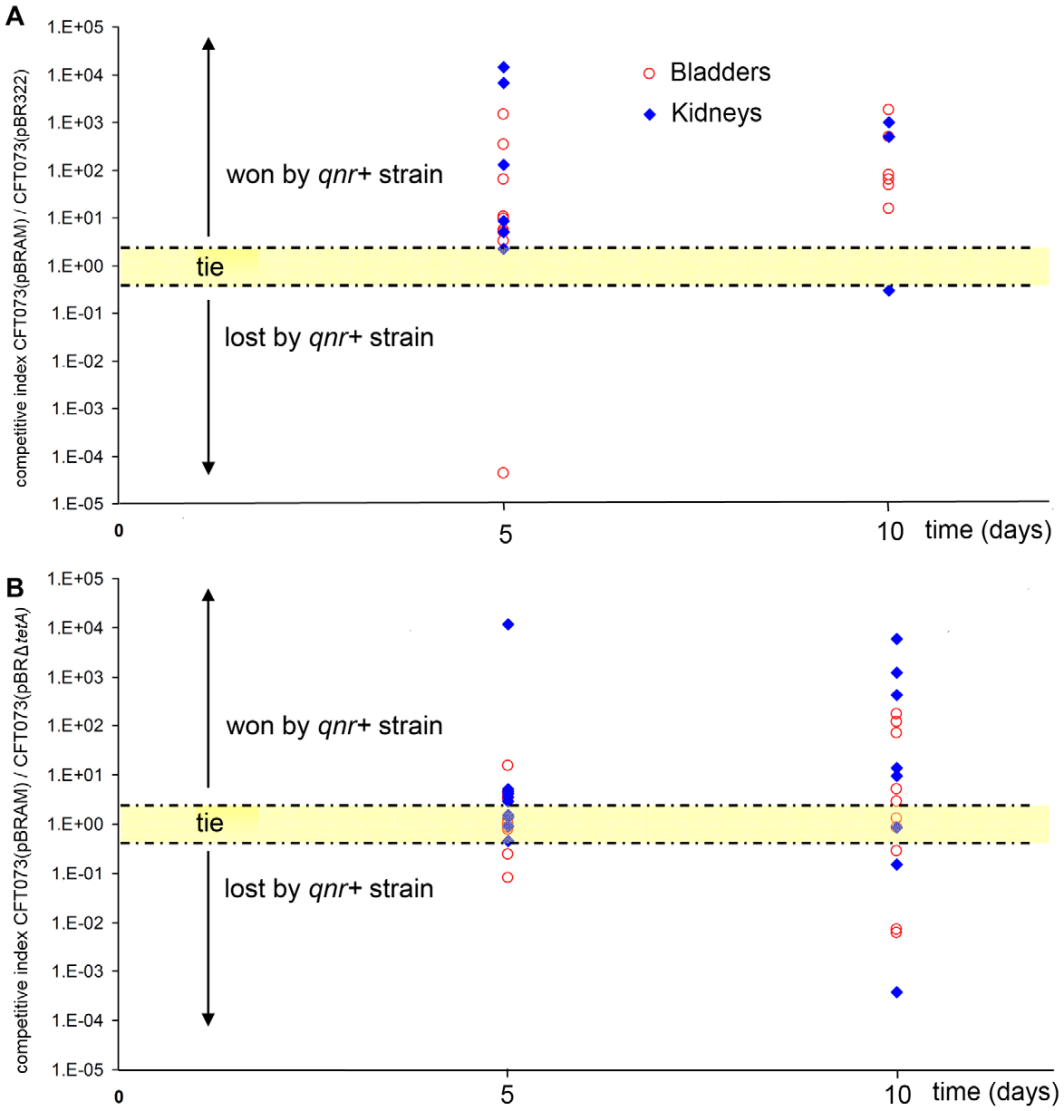
Enhanced fitness observed in competitive infections for *E. coli* CFT073 after *qnrA3* acquisition onto pBR322. Each symbol represents the bacterial ratio (number of CFU for the *qnr*-positive strain/number of CFU for the *qnr*-negative isogenic strain) measured in organs (blue diamond = kidneys, red circle = bladder) collected five and ten days after inoculation of a 1∶1 mix of the two strains. When the ratio was equal to 1+/−0.2, it was considered as tie. Part A: competitions experiments opposing *E. coli* CFT073(pBR322) (*qnr*−, *tetA*+) and *E. coli* CFT073(pBRAM2) (*qnrA3*+, *tetA*−). Fifteen mice were inoculated, 15 bladders and 10 pairs of kidneys were efficiently infected. Competition was won 22 times by *E. coli* CFT073(pBRAM2) (*qnrA3*+, *tetA*−), was lost 2 times, and one was tie (p<0.0001). Part B: competitions opposing *E. coli* CFT073(pBRΔ*tetA*) (*qnr*−, *tet*−) and *E. coli* CFT073(pBRAM2) (*qnrA3*+, *tet*−). Twenty-three mice were inoculated, 20 bladders and 17 pairs of kidneys were efficiently infected. Competition was won 24 times by *E. coli* CFT073(pBRAM2) (*qnrA3*+, *tetA*−), was lost 9 times, and 6 was tie (p<0.0001).

We performed *in vivo* experiments with the strain *E. coli* CFT073(pBRAM2) rather than with *E. coli* CFT073(pBRAM1) in order to be closer to clinical conditions (233-bp vs. 24-bp upstream sequence). In competitive infections (Figure 2A), *E. coli* CFT073(pBRAM2) (*qnrA3*+) had a significant advantage upon *E. coli* CFT073(pBR322) (*tetA*+) with 22 organs/25 where it had takeover versus only 2/25 where it had lost competition (p<0.0001). This selective advantage was confirmed when competitions were run against *E. coli* CFT073(pBRΔ*tetA*) (*qnr*−) (Figure 2B). Indeed among 37 competitions, 24 were won by the *qnrA3*-positive strain, 4 were judged as tie because the ratio of the two populations was 1 (+/−0.2) and 9 were lost (p = 0.01). At day 10, a median competitive index was calculated for *qnr*-positive strains as described previously [29]. It was 1.27+/−0.16 in bladders and 13.9+/−7.2 in kidneys.

### Stability of pBR322-derived plasmids in *in vitro* and *in vivo* experiments

*In vitro*, no plasmid loss was observed after daily culture of *E. coli* CFT073(pBR322), *E. coli* CFT073(pBRAM2) and *E. coli* CFT073(pBRΔtetA). *In vivo*, the mean loss of the plasmids pBR322 and pBRAM2 in single strain infections (data not shown) was 47% and 67%, respectively, which was not statistically different. In the competitive experiments, the global plasmid loss was higher when we used *E. coli* CFT073(pBR322) as a control (37% at day 5 and 80% at day 10) than when we used *E. coli* CFT073(pBRΔ*tetA*) (3% at day 5 and 11% at day 10). This suggests that the high plasmid loss observed in the competition with *tetA*-bearing cells corresponded mainly to the loss of pBR322.

In the competitive assays where *E. coli* CFT073(pBRΔtetA) was used for control, among 45 organs studied, 4 (9%) organs showed only ampicillin-susceptible colonies corresponding to a plasmid loss of 100%. When *E. coli* CFT073(pBR322) was used as control, it was 10/35 (29%) organs showing only ampicillin-susceptible colonies. Although we excluded these organs in the final counting, it showed that pBR322-derived plasmids were more stable *in vivo* when they carried a *qnrA3* gene that when they did not (p = 0.038), even considering the biological cost of *tetA*.

### Fitness cost due to acquisition of conjugative multidrug resistance plasmids harboring *qnr*

Reduced growth capacity of *E. coli* after pHe96 acquisition suggested a fitness cost. This was confirmed by the results of the *in vitro* competitive assays (eight repeated experiments growing together *E. coli* CFT073-Sm^R^ and *E. coli* CFT073-Sm^R^(pHe96)) where relative fitness index (RF) of *E. coli* CFT073-Sm^R^(pHe96) was significantly less than 1 (0.67+/−0.17). The relative fitness of the “R42” variant was similar with an index of 0.68+/−0.14. Plasmid loss measured for *E. coli* CFT073-Sm^R^(pHe96) was 65% after 30 generations. In contrast, plasmid loss was only 3% for its variant isolate R42 suggesting a compensatory mutation in the chromosome or the plasmid. To investigate whether the reduced growth observed with pHe96 exists for other *qnr* genes and other *qnr*-positive MDR plasmids, we studied the *in vitro* growth capacity of *E. coli* J53 and of its transconjugants carrying five different *qnr*-positive and MDR plasmids previously described [13], [35], [37]: pHm13 (*qnrA1*), pHm477 (*qnrA1*), pHe96 (*qnrA3*), pPS105 (*qnrS1*), and pU1696 (*qnrB4*). No difference was seen between growth parameters of the strain *E. coli* J53 and those of the five *qnr*-positive transconjugants (Table S1).

**Figure 3:**
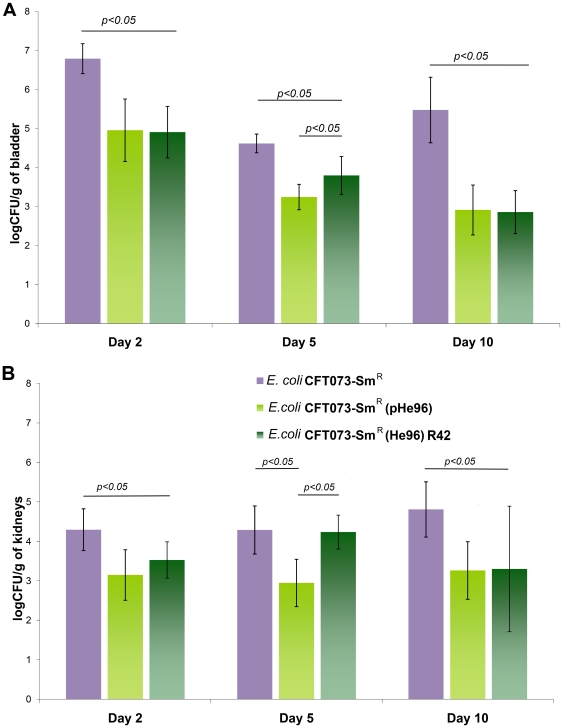
Single strain urinary tract infections with the isogenic system of *E. coli* CFT073-SmR harboring or not the multidrug resistance plasmid pHe96 (*qnrA3*). Part A: Bacterial density (log_10_CFU/g of tissue) in bladders collected two, five and ten days after inoculation by *E. coli* CFT073-Sm^R^ (*qnr*−, purple plot), *E. coli* CFT073-Sm^R^(pHe96) (*qnrA3*+, light green plot) and *E. coli* CFT073-Sm^R^(pHe96) R42 variant (*qnrA3*+, dark green plot). At day 2, bacterial density was 6.79+/−0.35, 4.95+/−0.8 (p<0.0001), and 4.9+/−0.65 (p<0.0001), respectively; at day 5, it was 4.61+/−0.25, 3.24+/−0.3 (p<0.0001) and 3.79+/−0.5 (p = 0.03), respectively; and at day 10, 5.47+/−0.8, 2.91+/−0.6 (p = 0.0004) and 2.85+/−0.5 (p = 0.001), respectively. At least 10 mice were studied per group. Part B : Bacterial density (log_10_CFU/g of tissue) in kidneys collected two, five and ten days after inoculation by *E. coli* CFT073-Sm^R^ (*qnr*−, purple plot), *E. coli* CFT073-Sm^R^(pHe96) (*qnrA3*+, light green plot) and *E. coli* CFT073-Sm^R^(pHe96) R42 variant (*qnrA3*+, dark green plot). Results were respectively 4.29+/−0.53, 3.14+/−0.64 (p = 0.005) and 3.53+/−0.46 (p = 0.053) at day 2; 4.29+/−0.61, 2.94+/−0.6 (p = 0.02), and 4.23+/−0.43 (p = 0.06) at day 5; and 4.81+/−0.6, 3.26+/0.63 (p = 0.012 and 3.3+/−1.59 (p = 0.04) at day 10. At least 10 mice were studied per group.

**Figure 4:**
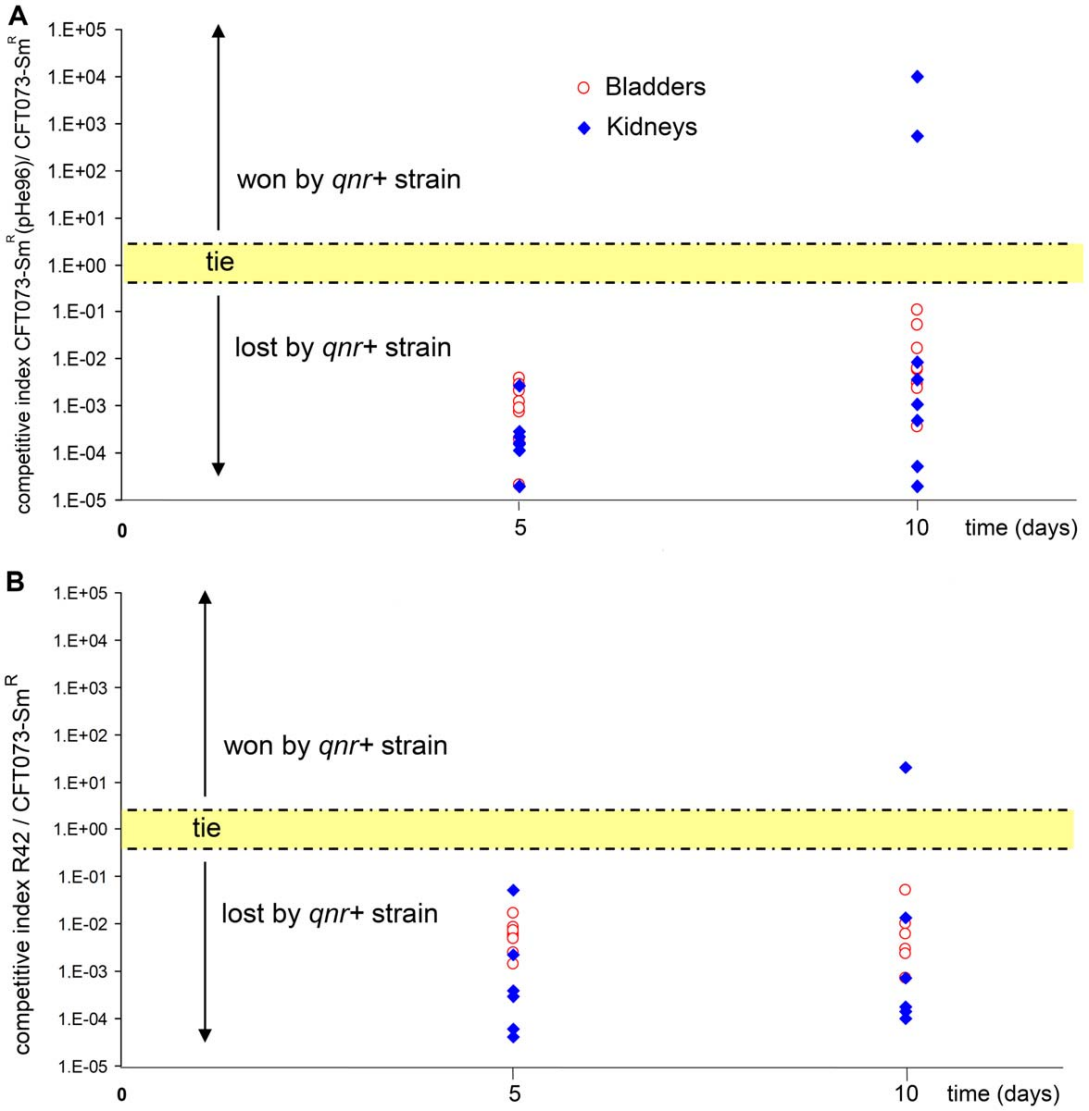
Reduced fitness observed after pHe96 acquisition in competitive infections in absence of antimicrobial exposure. Each symbol represents the ratio (number of CFU for the *qnr*-positive strain/number of CFU for the *qnr*-negative isogenic strain) in organs (blue diamond = kidneys, red circle = bladder), collected five and ten days after inoculation of a 1∶1 mix of the two strains. Part A: competition experiments opposing *E. coli* CFT073-Sm^R^ (*qnr*−) and *E. coli* CFT073-Sm^R^(pHe96) (*qnrA3*+). Twenty mice were inoculated, 19 bladders and 16 pairs of kidneys were efficiently infected. Competition was lost 33 times by *E. coli* CFT073-Sm^R^(pHe96) (*qnrA3*+), and won only 2 times (p<0.0001). Part B: competition experiments opposing *E. coli* CFT073-Sm^R^ (*qnr*−) and *E. coli* CFT073-Sm^R^(pHe96) variant “R42” (*qnrA3*+). The R42 variant was selected from kidneys that were infected by *E. coli* CFT073-Sm^R^(pHe96). Twenty mice were inoculated, 18 bladders and 16 pairs of kidneys were efficiently infected. Competition was lost 33 times by the *qnrA3*-positive strain with only one won (p<0.0001).

When mice were inoculated by the strain *E. coli* CFT073-Sm^R^ (pHe96), the bacterial density in bladders and kidneys was significantly lower than with the control strain, either at day 2, day 5 or day 10 (Figure 3). In addition, the number of mice where the strain failed to induce a persistent UTI was higher with the transconjugant than with the parental strain: 3 versus 0 for bladders and 7 versus 2 for kidneys (p = 0.003). The results were similar for the “R42” variant carrying the plasmid pHe96 but less than with the original transconjugant. This suggests that a compensatory mutation might have occurred in this variant when it was cultivated into the mouse for the first passage. In competitive experiments, *E. coli* CFT073-Sm^R^(pHe96) lost the competition in 33 out of 35 organs (p<0.05) with regard to the control strain *E. coli* CFT073-Sm^R^ confirming the fitness cost of pHe96 acquisition (Figure 4A). A similar rate of lost experiments was found for the “R42” variant (Figure 4B). The mean loss of pHe96 was less than 20% for the strains *E. coli* CFT073-Sm^R^(pHe96) and its variant “R42”, which was expected since these plasmids were coming from clinical isolates. The median value of the competitive index for the transconjugants carrying pHe96 was 0.0012+/−0.001. No competition has been run opposing the two pHe96 transconjugants because it was not possible to distinguish them.

We also tested, in competitive experiments against the *E. coli* CFT073 strain and using the same UTI mouse model, two *qnr*-positive uropathogenic clinical isolates: *E. coli* Hm13 (*qnrA1*) and *E. coli* PS105 (*qnrS1*) (Figure S2). In these non-isogenic competitions, the susceptible strain *E. coli* CFT073 won 53 of 57 competitions (kidneys and bladders) upon *E. coli* Hm13 (*qnrA1*) and 40 of 65 competitions upon *E. coli* PS105 (*qnrS1*) (p<0.001). This confirmed that the fitness cost attributed to the acquisition of pHe96 was not specific to this plasmid but was similarly observed with other *qnr*-positive MDR plasmids.

## Discussion

The interplay between resistance and fitness is a challenging issue when increase of antibiotic resistance is observed worldwide [26]. Selection pressure due to the increase in antibiotic prescription explains the increase in resistance in most of the settings [3] and quinolone resistance was so far associated to a fitness cost when it was due to mutations either in the topoisomerase genes or in the efflux operons [27]–[29], [45]–[49]. The emergence of plasmid-mediated resistance determinants questioned about links between acquisition of resistance and selective advantage. Although plasmid acquisition brings a fitness cost, the stability of native plasmids varied according to the host and the presence of drug addiction systems may compensate this plasmid cost [50], [51]. For some antibiotic resistance determinants described recently, such as CTX-M beta-lactamases [52], [53], we know that they have been transferred from environmental bacteria harboring the resistance genes as chromosomal-borne, to human commensal bacteria, mainly *E. coli*, through mobile elements such as plasmids [54]. For quinolone resistance, such transfer may have occurred since similar *qnr* genes are chromosome-borne in environmental bacteria [18]. However, the persistence of *qnr* genes on plasmids is not fully explained by the quinolone selective pressure since there are numerous other effective mechanisms to obtain quinolone resistance such as stepwise chromosomal mutations in the target genes [55] and in the several efflux systems present in *E. coli* [56]. We hypothesized that *qnr* genes confer a selective advantage outside the quinolone exposure.

To study the impact on fitness of *qnr* acquisition, we compared *in vitro* growth curves, *in vitro* pairwise competition, and *in vivo* single culture and pairwise competition, which are usual methods found in literature [26], [28], [29], [42]–[45], [57]. Pairwise competitions are usually more sensitive than single cultures to reveal a fitness change, and *in vivo* assays are more relevant than *in vitro* assays since the complex growth environment is closer to reality [26]. This justifies the conclusions we drew on *qnr* impact on fitness, which were based on the results of *in vivo* competitions assays. We chose the mouse model of pyelonephritis for *in vivo* experiments because it was well validated and recently used for studying the interplay in fluoroquinolone resistance mutations and bacterial fitness [29]. Marcusson and colleagues showed that although the first quinolone resistance mutations (mostly *gyrA*) had fitness cost, latest compensatory *parC* mutations could provide increase of both resistance and fitness, suggesting that a higher level of resistance could be selected in absence of antimicrobial exposure [29].

Our findings, obtained using an isogenic system in *E. coli* where the *qnr* gene was cloned onto a simple replicative plasmid such as pBR322, showed that *qnr* acquisition enhanced the bacterial fitness *in vitro* and *in vivo*. Indeed when the only difference between two *E. coli* strains was the presence of *qnr* with its flanking region, the strain that acquired the *qnr* gene took over the susceptible strain in absence of quinolone exposure. Several studies [13], [14] suggested that the low level quinolone resistance is relevant when the host is exposed to quinolone in clinical situations. This could give a partial explanation for widespread but not for emergence. Several environment bacterial species, such as *Shewanella algae* or *Vibrio splendidus*, have been shown to harbor chromosomal *qnr*-like genes and are supposed to be the reservoir of *qnrA3* and *qnrS1*, respectively [15]–[18]. The enhancement of fitness we observed after the acquisition by *E. coli* CFT073 of *qnrA3* without antimicrobial exposure could explain how *qnr* had first emerged. This could have been the following scenario: the transfer from the chromosome of *S. algae* to a mobile element replicating in *E. coli*, or another member of the *Enterobacteriaceae* family, due to *qnr* giving a selective advantage. *qnrD* is the last *qnr* gene that was discovered in 2009 and it is carried onto a small non conjugative plasmid [58]. Although we did not test the fitness conferred by this small replicative *qnr*-positive plasmid, similar to pBR322, we hypothesized that the first step of the *qnr* emergence could have been through mobilization onto small plasmids, which may have resulted in an enhanced fitness as we showed in our *in vivo* experiments. In addition, there might be low concentrations of quinolones in the environment at some places which could have contributed to the resilience of these bacteria harboring *qnr* genes [59].

To investigate if their impact on fitness could also explain the widespread of *qnr* genes, we measured the fitness after acquisition of *qnrA3* when included in a multidrug resistant plasmid as those usually observed in clinical *qnr*-positive isolates [10], [11], [36], [60]. When the gene was acquired with the whole multi drug resistant plasmid pHe96, the host fitness was lessened. This could be explained by the presence of another fitness impairing gene on the plasmid. However, this fitness cost was not specific of pHe96 since competitive experiments between *E. coli* CFT073 and two other *E. coli* clinical strains with *qnr* containing MDR plasmids showed the same fitness cost. Our experiments with an evolved clone of the *E. coli* CFT073(pHe96) transconjugant suggest that the fitness cost can be reduced by the host. Further explorations are needed to investigate the mechanism of this compensation. The difference in the impact on fitness when *qnr* is included into a large plasmid or into a small non conjugative plasmid could also be a consequence of a different level of *qnr* expression. However, MICs of quinolones were the same in the two plasmids.

Whatsoever, this would mean that fitness gain could contribute to the emergence of qnr-bearing plasmids among the bacterial communities living in the environment (soil, water) and their transfer to other bacteria such as those of the human commensal flora [54]. For the widespread observed in the following years, the fluoroquinolone resistance conferred and co selection with other resistance determinant (particularly expanded-spectrum-beta-lactamases, ESBL) would be the predominant selection factor [61], [62].

Native functions of Qnr proteins are still unknown. They bind type II topoisomerases even without quinolone binding to their targets [19], [20]. Although known Qnr proteins usually do not interfere with *E. coli* gyrase activity at the concentration they prevent fluoroquinolone inhibition [10], [19], [21], Qnr-like proteins inhibit catalytic activity of topoisomerases from their natural host [21], [63]. Crystallographic data on the structure of Qnr-like PRPs such as MfpA [22], *Efs*Qnr [23] and AhQnr [24], revealed a right handed β helix structure that mimics a double-strain helix DNA. This could permit interaction with other DNA-linked proteins with a possible regulating function of the activity of these proteins. Mimicking DNA structure could have consequences on cell cycle and replication. Thus, this could explain the impact on fitness.

### Limitations

Plasmid instability was significant for some of our plasmid constructions, even the native plasmid pHe96. However our analysis took into account this plasmid loss. Overall, artificial plasmids constructed from pBR322 were less stable than plasmids naturally isolated from clinical strains.

## Materials and Methods

### Ethics Statement

Animal experiments were performed in accordance with prevailing regulations regarding the care and use of laboratory animals by the European Commission. The experimental protocol was approved by the Departmental Direction of Veterinary Services in Paris, France (agreement N° A 75-18-05, 2005). The procedures are conformed to the Amsterdam protocol on animal protection and welfare and Directive 86/609/EEC on the protection of animals used for experimental and other scientific purposes.

All manipulation of animals were be carried out by qualified personnel. All protocols will be done in accordance with the local institutional review board (Comité d'Ethique en expérimentation animale, Ile de France, Paris Comité 3).

### Bacterial strains and plasmids

*E. coli* CFT073 strain is a clinical isolate susceptible to all antibiotics [32], which was used previously to set the murine model of pyelonephritis [13], [33]. The plasmid pBR322 vector (4,361 bp) carries a *bla* gene encoding ampicillin resistance and a *tetA* gene encoding tetracycline resistance [64], [65]. The plasmid pHe96 was previously isolated in a *Klebsiella pneumoniae* cultivated from feces in a patient with cancer. The strain was described before [16] and the plasmid was sequenced for the 10 kb DNA fragment flanking *qnrA3* (GenBank accession number EU495237). *qnrA3* is a gene homologous to the chromosome-borne *qnr* allele found in *S. algae* strains [18] and was only described as plasmid-borne in the plasmid pHe96 [16].

*E. coli* J53 strain is a recipient strain widely used for conjugation with plasmid carrying *qnr* alleles [6], [36]. *E. coli* J53 transconjugants were obtained after conjugation with the following clinical strains: *E. coli* pHm477 (*qnrA1*) [11], *K. pneumoniae* pHe96 (*qnrA3*) [16], *E. coli* pPS105 (*qnrS1*) [13], *E. coli* pHm13 (*qnr A1*) [13] and *E. coli* pU1696 (*qnrB4*) [37]. These two latter strains, which were uropathogenic clinical isolates harboring multi drug resistance plasmids containing *qnrA1* and *qnrS1*, respectively, were used for competitive experiments against susceptible *E. coli* CFT073.

### *qnrA3* cloning into pBR322

Cloning of the *qnrA3* gene and its flanking region was done including either the 24 nucleotides (M24) upstream from *qnrA3*, resulting in the plasmid pBRAM1, or the 233 nucleotides (M233) upstream, resulting in the plasmid pBRAM2, and 29 nucleotides downstream for both. These upstream regions M24 and M233 are known to be conserved in *qnrA* containing plasmids [16] and are supposed to contain the promoter region [36], [66]. Amplification was performed using a sense primer containing an *EcoR*V restriction site (underlined) (5′-ATACTTCCGATATCCCCCTCCCTGATT-3′ for the fragment included in pBRAM1 and 5′-CTGACTGATATCCCCAAATCCAACACT-3′ for the fragment included in pBRAM2) and a *Sal*I restriction site (underlined) in the reverse primer (GCAAGCGTCGACTCAAGTGATATTTGC for both DNA amplified fragments). Plasmid DNA of *K. pneumoniae* He96 was used as the template. PCR was performed in a 25 µl mixture including 10 µl forward and reverse primers, 5 µl DNA template and one Illustra™ pure Taq™ Ready-To-Go™ PCR bead (GE Healthcare, Buckinghamshire, UK). PCR was run using the following program : 94°C for 4 min; 94°C for 1 min; 55°C for 1 min; 72°C for 1 min for 30 cycles; and 72°C for 10 min. The amplified fragment was purified using Qiaquick® PCR purification kit (Qiagen®), digested with *EcoR*V and *Sal*I (Invitrogen™), and then ligated into the tetracycline resistance *tetA* gene downstream of the promoter of pBR322 (Invitrogen™) previously digested with *EcoR*V and *Sal*I. The ligation product was electroporated into competent *E. coli* CFT073. Transformants for *E. coli* CFT073 with pBRAM1 or pBRAM2 were selected on ampicillin (100 µg/ml)-containing Mueller-Hinton (MH) plates and replicated on tetracycline (20 µg/ml)-containing plates to identify inserts.

The control strains were *E. coli* CFT073(pBR322), where the plasmid pBR322 was electroporated into competent *E. coli* CFT073, and *E. coli* CFT073(pBRΔ*tetA*) where the *tetA* gene in the vector pBR322 was inactivated as follows: pBR322 was digested with *EcoR*V and *Nru*I, ligated without any insert, and the product was electroporated in competent *E. coli* CFT073. Susceptibility to tetracycline was tested to check *tetA* inactivation. Figure S1 summarizes in a scheme the methods used to obtain the pBR322 derivatives.

### pHe96 containing strains and controls

A streptomycin-resistant mutant *E. coli* CFT073-Sm^R^ strain was selected *in vitro* from *E. coli* CFT073 by plating 10^9^ bacteria onto MH agar containing streptomycin concentration from 10 to 160 µg/ml. The transconjugant *E. coli* CFT073-Sm^R^(pHe96) was obtained after conjugation between *E. coli* CFT073-Sm^R^ and *K. pneumoniae* He96 [16] after 40 min of mating into MH broth as previously described [13]. After incubation, transconjugants were selected by plating the conjugation mixture on MH agar supplemented with ampicillin (100 µg/ml) plus streptomycin (100 µg/ml). PCR experiments (RAPD and PCR *qnrA*) were performed as already described to confirm the success of the conjugation [11], [67].

*E. coli* CFT073-Sm^R^(pHe96) “R42” is a clone of *E. coli* CFT073-Sm^R^(pHe96), which has been selected from kidneys of an infected mouse. This clone was studied because it caused an infection with a bacterial load in organs higher than with the other clones of *E. coli* CFT073-Sm^R^(pHe96).

### Growth experiments and antibiotic susceptibility testing

Growth curves of each strain in TS broth were drawn using automatic optical density measurements obtained by reader infinite® M200 (Tecan). After an overnight culture in TS broth at 37°C, bacterial suspensions were diluted in order to reach an Optical Density of 0.5, and then diluted 100 times. Microplates were inoculated with the obtained suspensions. Each combination of strain and medium was run in triplicate. The plates were incubated at 37°C for 24 hours and subject to shaking during 60 s every two minutes. Optical density (OD) at 600 nm was measured every 5 minutes during this 24-hours period. The log_10_ of each value of OD was calculated. To estimate the doubling time, a slope was calculated between every two consecutive values. After discarding artifacts, a mean of the six highest slopes was calculated, corresponding to the maximal growth rate. Doubling time was calculated as log_10_ 2 divided by the maximal growth rate [57], [68]. Maximal OD increase was also calculated as the mean of the ten highest values of OD reached during stationary phase. This has been performed five times for each strain.

The Minimal Inhibitory Concentrations (MICs) of nalidixic acid, norfloxacin, ofloxacin and ciprofloxacin were determined for each constructed strain and control strain by Etest®, according to the instructions of the manufacturer. MICs of amikacin and tobramycin were also measured in order to evaluate the expression of *aac(6′)-Ib-cr*. Etest® were performed on MH agar plates and the plates were incubated 18 h at 37°C.

### Plasmid stability

Since all the plasmids tested carried an ampicillin resistant determinant, plasmid stability of the transconjugants *E. coli* CFT073-Sm^R^(pHe96), *E. coli* CFT073(pBRAM1), *E. coli* CFT073(pBRAM2), *E. coli* CFT073(pBR322) and *E. coli* CFT073(pBRΔ*tetA*) was measured *in vitro* by daily subculture during ten days in antibiotic-free Trypticase Soy (TS) broth with plating onto TS agar containing 100 µg/ml ampicillin and antibiotic free TS agar plates. In vivo, at the end of each experiment, organs were also spread onto ampicillin-containing and antibiotic-free TS agar. Plasmid loss was calculated for each organ as the ratio ampicillin resistant CFU/total number of CFU.

### Flow cytometry

In order to check that OD variations were not caused by a change of cell size, all the studied strains were also studied in flow cytometry as already described [69]. Flow cytometric measurements were performed on a Guava EasyCyte Plus flow cytometer (Millipore, St Quentin en Yvelines, France) equipped with a 488 nm laser. Overnight cultures were diluted to a cell density of ca. 10^5^ CFU/ml. Cells were collected on the forward scatter with logarithmic amplifiers for 5,000 events. The experiment was replicated four times for each strain.

### In vitro competitions experiments

Pairwise competition experiments were performed to evaluate the in vitro relative fitness of each *qnr*-positive strain compared to the *qnr*-negative control strain. We also performed competition with the wild type strain *E.coli* CFT073. Each strain of its isogenic system was incubated separately for 18 h at 37°C in TS broth. The cell densities of the suspensions were estimated by flow cytometry [70]. An aliquot containing 10^8^ bacteria was taken from each suspension and mixed two by two in a ratio of 1∶1 to inoculate 10 ml of TS broth and then incubated for 24 h at 37°C for a competitive growth. The initial and the final densities of each competitive strain were estimated from CFU data by diluting and plating population samples onto TS agar with and without antibiotic. Tetracycline was used to distinguish *E. coli* CFT073(pBR322) from *E. coli* CFT073(pBRAM1) and *E. coli* CFT073(pBRAM2) in the first isogenic system Ampicillin was used to distinguish *E. coli* CFT073 from *E. coli* CFT073(pBRAM1) and *E. coli* CFT073(pBRAM2). In the second isogenic system, ampicillin was used to distinguish *E. coli* CFT073-Sm^R^(pHe96) from *E. coli* CFT073-Sm^R^. With these densities, the Malthusian parameter of each competitor could be calculated. The Relative Fitness (RF) was calculated as the ratio of the two Malthusian parameters [42]–[45] (RF = (logS1_d1_−logS1_d0_)/log (S2_d1_−logS2_d0_), where S1_d1_ and S1_d0_ are the CFU densities of the *qnr+* strain respectively at the end and at the beginning of the competition experiment, and S2_d1_ and S2_d0_ their equivalent values for the control strain. A RF score >1 meant that the *qnr+* strain had a selective advantage upon the control strain whereas a score <1 represented a fitness cost for *qnr* acquisition.

### Mouse model of urinary tract infection

The ascending unobstructed mouse model of UTI was used as previously described [13], [33]. Animal experiments were performed in accordance with prevailing regulations regarding the care and use of laboratory animals by the European Commission. The experimental protocol was approved by the Departmental Direction of Veterinary Services in Paris, France. Eight-week-old immunocompetent female CBA mice (weight, 20 to 23 g) were used. Inocula of different strains were obtained by overnight incubation in BHI broth, washing of the cells by centrifugation at 15,000× *g* for 15 min, and resuspension in TS broth to a final inoculum of 10^10^ CFU/ml. Pyelonephritis was induced during general anaesthesia (with an intraperitoneal administration of Ketamin 150 mg/kg and Xylazin 0.5 mg/kg) by injecting 50 µl (i.e., 5×10^8^ CFU) into the bladder through a urethral catheter.

### In vivo growth experiments: single-strain infections

In order to evaluate their potentials to perform persistent UTI and to reach a high bacterial density in bladder and kidneys, mice were inoculated separately by each of the following strains: *E. coli* CFT073-Sm^R^, *E. coli* CFT073-Sm^R^(pHe96) and *E. coli* CFT073-Sm^R^(pHe96) “R42”. No antimicrobial was administrated. Mice were sacrificed at two, five and ten days after inoculation with at least 8 mice per group, and bladders and kidneys were aseptically taken out, weighed and homogenized in 1 ml of saline solution. Bacterial densities were estimated from CFU data by diluting and plating organs samples onto TS agar and were expressed as the number of CFU/g of tissue. The growth rate of each strain was estimated by the bacterial densities in bladder and kidneys and could be compared to the control strain. Plasmid loss on days 5 and 10 was assessed for strains harboring plasmid pHe96 by comparing the number of CFU growing onto MH plates containing or not ampicillin (100 µg/ml).

### In vivo fitness measurements: competitive infections

The *in vivo* selective value of the *qnr*-positive strain compared to the control *qnr*-negative strain was determined by competitive experiments between the two isogenic strains using the same mouse model of UTI. Inocula were prepared the same way that for single-strain infection experiments, except that before mixing the two suspensions, bacterial density was measured by flow cytometry. Proportions of each suspension were adjusted so that inoculum contained the two strains in a 1∶1 ratio and that cell density was at least 10^10^ bacteria/ml. Inoculum was spread on antibiotic containing plate to count the initial ratio. At least 20 mice were inoculated for each competitive experiment, and were sacrificed at five or ten days after inoculation. After removing and homogenizing bladders and kidneys, they were spread onto TS agar plates without antibiotics and containing the appropriate antibiotic to select one of the two competitive strains among the population. Tetracycline (20 µg/ml) was used to distinguish between strains harboring pBR322 or pBRAM2, and ciprofloxacin (0.05 µg/ml) to distinguish strains harboring pBRAM2 or pBRΔ*tet*A. To count the number of pBRΔ*tet*A-bearing cells, we subtracted the number of cells growing onto ciprofloxacin-containing plates from the number of cells growing onto ampicillin-containing plates. Ampicillin (100 µg/ml) was used to distinguish *E. coli* CFT073-Sm^R^ from its transconjugants, *E. coli* CFT073-Sm^R^(pHe96) and *E. coli* CFT073-Sm^R^(pHe96) R42. For competitions between strains carrying pBR322 or its derivatives, bladders and kidneys were also spread onto ampicillin-containing plates, in order to evaluate plasmid loss. A competitive index was calculated for each organ as the ratio of the number of *qnr*-positive cells/number of *qnr*-negative cells, corrected by the initial ratio. Organs where no CFU grew were excluded.

### Statistical analysis

Results were expressed as mean and 95% confidence interval for continuous variables. Continuous variables were compared by nonparametric (Wilcoxon) tests. Discontinuous variables were compared by Fisher's exact test. A *p* value less than 0.05 was considered significant. Analysis was performed using the online free software Biostatgv (http://www.u707.jussieu.fr/biostatgv).

## Supporting Information

Figure S1 **Scheme of** ***qnrA3*** **cloning from its native plasmid pHe96 into pBR322 and resulting** ***qnr*** **-positive (pBRAM1 and pBRAM2) and control plasmids (pBRΔtetA).** M24 and M233 are the fragments of 24- and 233-bp upstream from *qnrA3* and described in pHe96 [16]. Primers (see text for sequences) Fw2, Fw1 and R contain the *Eco*RV and *Sal*I restrictions sites and were used to amplify the *qnrA3* DNA fragments.
(TIF)

Figure S2 **Reduced fitness observed for clinical isolates of** ***E. coli*** **harboring** ***qnr*** **-positive multi drug resistance plasmids in competitive infections with** ***E. coli*** **CFT073 without antimicrobial exposure.** Each symbol represents the ratio (number of CFU for the *qnr*-positive strain/number of CFU for the *qnr*-negative isogenic strain) in organs (blue diamond = kidneys, red circle = bladder), collected five to forty days after inoculation of a 1∶1 mix of the two strains. Part A: competition experiments opposing *E. coli* CFT073 (*qnr*−) and *E. coli* Hm13 (*qnrA1*+). Thirty mice were inoculated, 27 bladders and 30 pairs of kidneys were efficiently infected. Fifty-three competitions were lost by the *qnr*+ strain and 2 only were won (p<0.0001). Part B: competition experiments opposing *E. coli* CFT073 (*qnr*−) and *E. coli* PS105 (*qnrS1*+). Thirty-three mice were inoculated, 33 bladders and 30 pairs of kidneys were efficiently infected. Forty-one competitions were lost by the *qnr*+ strain, and 17 were won (p<0.0001).
(TIF)

Table S1 *In vitro* growth parameters of *E. coli* J53 and its transconjugants with *qnr*-positive multidrug resistant plasmids.
(DOC)
