# Supplementary figures and images for: Plasmidic qnrA3 Enhances Escherichia coli Fitness in Absence of Antibiotic Exposure
Source: PLoS One. 2011 Sep 7;6(9):e24552. doi: 10.1371/journal.pone.0024552 (PMC3168526; doi:10.1371/journal.pone.0024552)

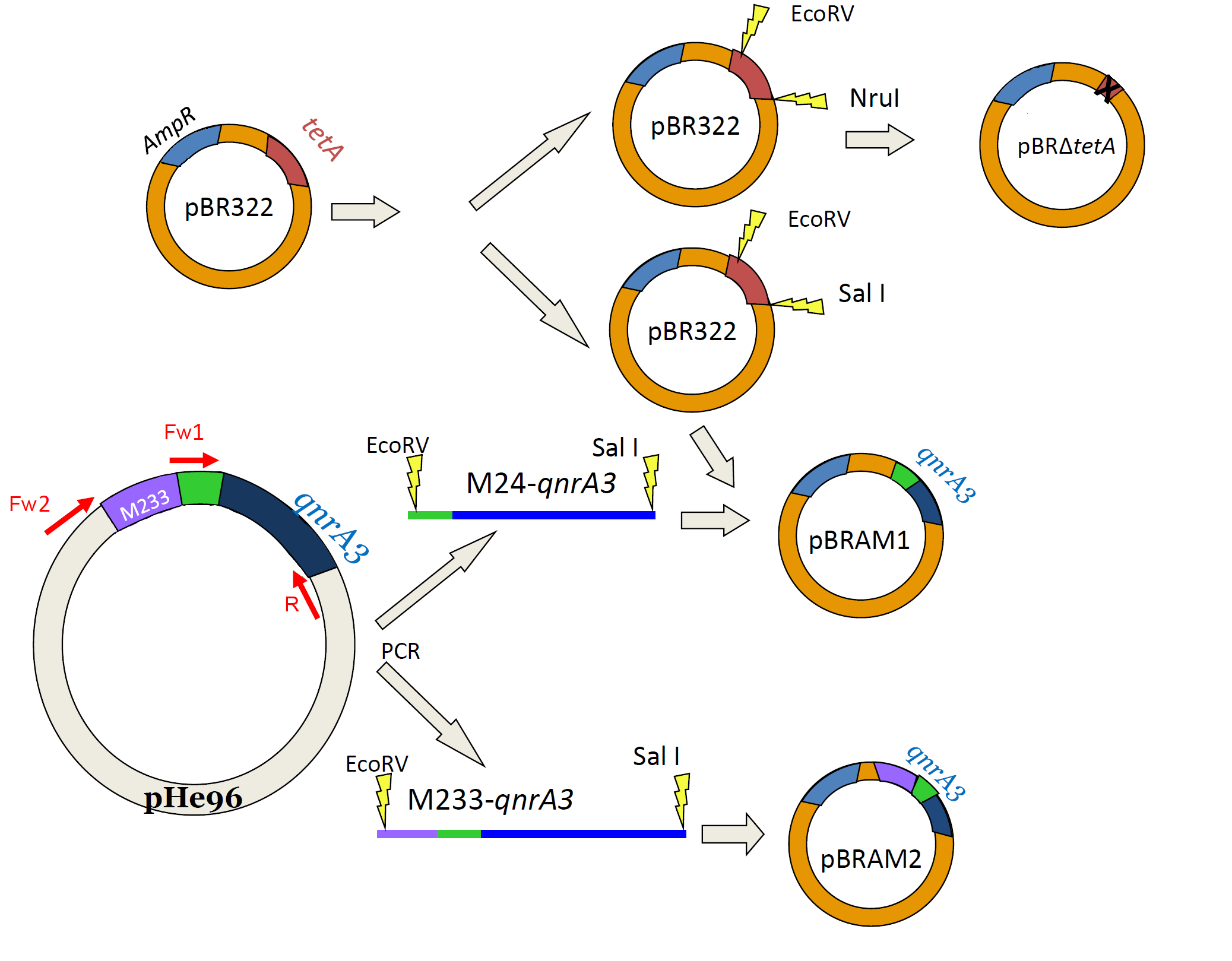

Supplement: Figure S1 — Scheme of qnrA3 cloning from its native plasmid pHe96 into pBR322 and resulting qnr -positive (pBRAM1 and pBRAM2) and control plasmids (pBRΔtetA). M24 and M233 are the fragments of 24- and 233-bp upstream from qnrA3 and described in pHe96 [16]. Primers (see text for sequences) Fw2, Fw1 and R contain the EcoRV and SalI restrictions sites and were used to amplify the qnrA3 DNA fragments. (TIF) [file pone.0024552.s001.tif]

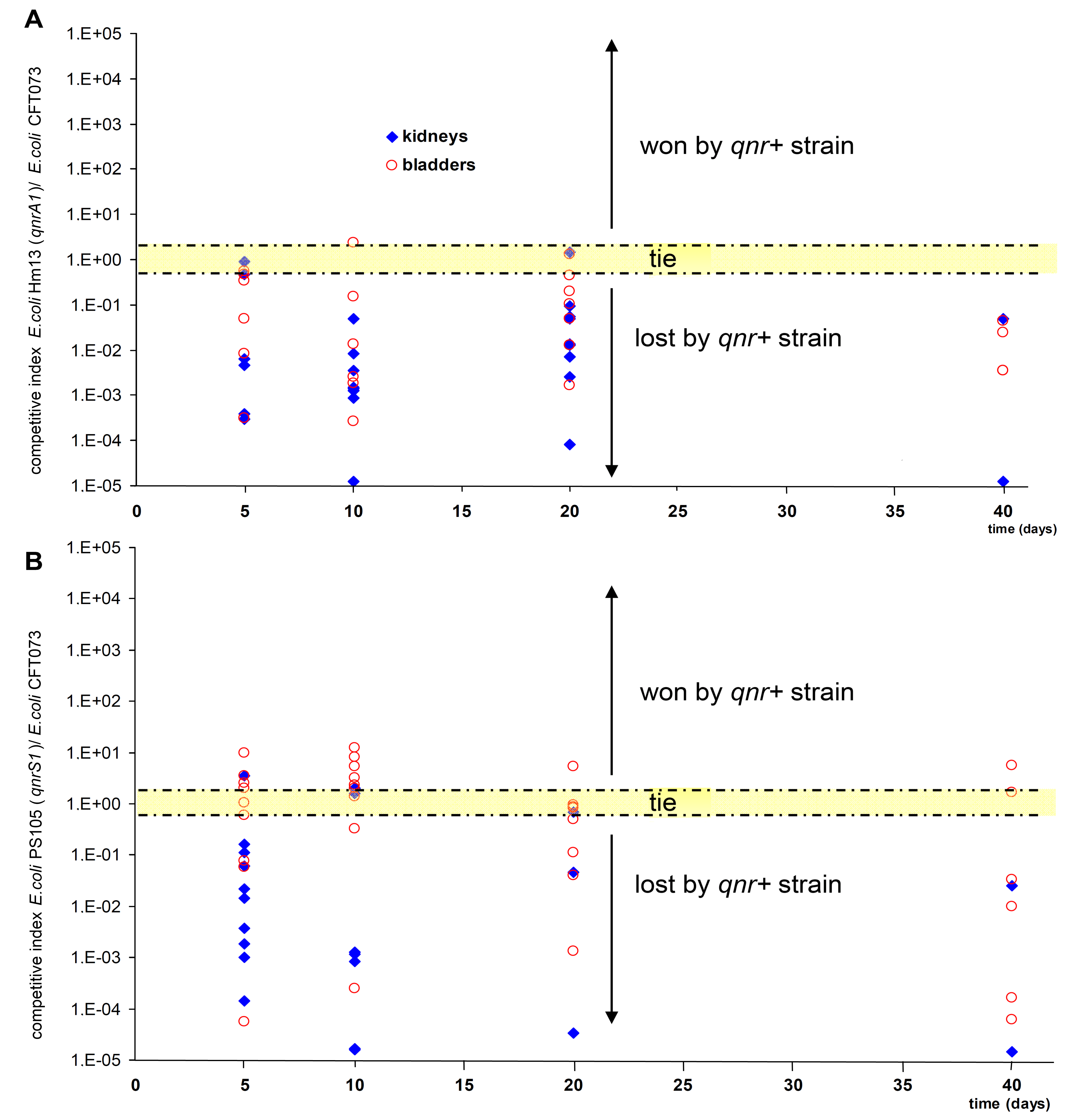

Supplement: Figure S2 — Reduced fitness observed for clinical isolates of E. coli harboring qnr -positive multi drug resistance plasmids in competitive infections with E. coli CFT073 without antimicrobial exposure. Each symbol represents the ratio (number of CFU for the qnr-positive strain/number of CFU for the qnr-negative isogenic strain) in organs (blue diamond = kidneys, red circle = bladder), collected five to forty days after inoculation of a 1∶1 mix of the two strains. Part A: competition experiments opposing E. coli CFT073 (qnr−) and E. coli Hm13 (qnrA1+). Thirty mice were inoculated, 27 bladders and 30 pairs of kidneys were efficiently infected. Fifty-three competitions were lost by the qnr+ strain and 2 only were won (p<0.0001). Part B: competition experiments opposing E. coli CFT073 (qnr−) and E. coli PS105 (qnrS1+). Thirty-three mice were inoculated, 33 bladders and 30 pairs of kidneys were efficiently infected. Forty-one competitions were lost by the qnr+ strain, and 17 were won (p<0.0001). (TIF) [file pone.0024552.s002.tif]
