# Supplementary material for: Plasmidic qnrA3 Enhances Escherichia coli Fitness in Absence of Antibiotic Exposure
Source: PLoS One. 2011 Sep 7;6(9):e24552. doi: 10.1371/journal.pone.0024552 (PMC3168526; doi:10.1371/journal.pone.0024552)
Supplement: Table S1 — In vitro growth parameters of E. coli J53 and its transconjugants with qnr-positive multidrug resistant plasmids. (DOC) [file pone.0024552.s003.doc]

**Table S1.** *In vitro* growth parameters of *E. coli* J53 and its transconjugants with *qnr*-positive multidrug resistant plasmids.

| ***E. coli* strains** | **Maximal growth rate in log(OD)/h.** | | **Doubling time in min** | | **Maximal OD** | |
| --- | --- | --- | --- | --- | --- | --- |
|  | **mean** | ***p*^a^** | **mean** |  | **mean** | ***p*^a^** |
| J53 | 0.69 (+/-0.03) | - | 26.2 (+/-1.3) | - | 1.17 (+/-0.04) | - |
| J53 Tc pHm13 (*qnrA1*) | 0.66 (+/-0.01) | 0.2 | 27.4 (+/-0.9) | 0.2 | 1.17 (+/-0.02) | 0.7 |
| J53 Tc pHm477 (*qnrA1*) | 0.71 (+/-0.05) | 0.95 | 25.5 (+/-1.0) | 0.95 | 1.12 (+/-0.04) | 0.1 |
| J53 Tc pHe96 (*qnrA3*) | 0.69 (+/-0.02) | 0.95 | 26.1 (+/-0.9) | 0.95 | 1.18 (+/-0.02) | 0.3 |
| J53 Tc pPS105 (*qnrS1*) | 0.68 (+/-0.02) | 0.6 | 26.5 (+/-1.0) | 0.6 | 1.16 (+/-0.02) | 0.8 |
| J53 Tc pU1696 (*qnrB4*) | 0.67 (+/-0.02) | 0.3 | 26.9 (+/-0.9) | 0.3 | 1.14 (+/-0.04) | 0.4 |

Data obtained from automatic OD measurements (n=15) in Trypticase Soy Broth and expressed as mean and Confidence Interval 95%.

^a^ Comparison with control strain (*qnr-*) performed with a Wilcoxon test. *p*. <0.05 was considered significant.
